# CO_2_ Hydrogenation on Metal-Organic Frameworks-Based Catalysts: A Mini Review

**DOI:** 10.3389/fchem.2022.956223

**Published:** 2022-07-18

**Authors:** Qian Zhang, Sen Wang, Mei Dong, Weibin Fan

**Affiliations:** ^1^ State Key Laboratory of Coal Conversion, Institute of Coal Chemistry, Chinese Academy of Sciences, Taiyuan, China; ^2^ University of the Chinese Academy of Sciences, Beijing, China

**Keywords:** MOFs, CO_2_ hydrogenation, methanol, methane, C2+ Products

## Abstract

Conversion of carbon dioxide (CO_2_) into value-added fuels and chemicals can not only reduce the emission amount of CO_2_ in the atmosphere and alleviate the greenhouse effect but also realize carbon recycling. Through hydrogenation with renewable hydrogen (H_2_), CO_2_ can be transformed into various hydrocarbons and oxygenates, including methanol, ethanol, methane and light olefins, etc. Recently, metal-organic frameworks (MOFs) have attracted extensive attention in the fields of adsorption, gas separation, and catalysis due to their high surface area, abundant metal sites, and tunable metal-support interface interaction. In CO_2_ hydrogenation, MOFs are regarded as important supports or sacrificed precursors for the preparation of high-efficient catalysts, which can uniformly disperse metal nanoparticles (NPs) and enhance the interaction between metal and support to prevent sintering and aggregation of active metal species. This work summarizes the recent process on hydrogenation of CO_2_ to methanol, methane and other C_2+_ products over various MOFs-based catalysts, and it will provide some dues for the design of MOFs materials in energy-efficient conversion and utilization.

## Introduction

Due to the rapid consumption of fossil resources, e.g., coal, petroleum, and natural gas, a large number of CO_2_ have been released into the atmosphere (Song, 2006). From 2006 to 2021, the global CO_2_ concentration in the atmosphere has been elevated from 381 to 415 ppm (NOAA, 2022). The massive emission of CO_2_ has brought serious environmental problems, such as global climate change and ocean acidification (Valles-Regino et al., 2015). Hence, reduction of CO_2_ amount and mitigation of greenhouse effect are the major challenges faced by the whole human society.

Regardless of this, CO_2_ is an important C1 platform molecule. Conversion of CO_2_ through sustainable catalytic processes into valuable chemicals and clean fuels is a promising way for CO_2_ utilization which could promote a circular carbon economy (Srinivas et al., 2014; Didas et al., 2015; Porosoff et al., 2016; Rafiee et al., 2018). CO_2_ conversion can be achieved by electro-catalysis, photo-catalysis, and thermal-catalysis processes. Electro-catalysis or photo-catalysis from clean and renewable electrical or solar energy is regarded as an important route for CO_2_ reduction reaction (CO_2_ RR). Through the rational design of high efficient catalysts, these reactions can be performed under relatively mild conditions that considerably decrease the energy consumption (Liu et al., 2012; Handoko et al., 2013; Jhong et al., 2013; Wang et al., 2015; Perathoner and Centi, 2019; Wang J.-J. et al., 2021; Zhang et al., 2021; Zhang et al., 2022a). Nevertheless, the electro-catalysis or photo-catalysis for CO_2_ conversion is time or geographically dependent, which, thus, decreases their economic viability. Compared to the former two manners, the thermal catalytic conversion of CO_2_ shows higher efficiency and it is more potential for industrial application. Since the CO_2_ molecule is thermodynamically stable and kinetically inert, the activation of the C=O bond in CO_2_ needs to overcome a high energy barrier. Renewable hydrogen (H_2_) generated from photolysis or electrolysis of water has high energy density and it can effectively reduce CO_2_. Thus, hydrogenation of CO_2_ into high-value hydrocarbons and oxygenates, including methane (CH_4_), methanol (CH_3_OH), ethanol (C_2_H_5_OH), and light olefins (C_2_^=^-C_4_^=^), has received increasing research interest (Sha et al., 2020).

The catalytic system for CO_2_ hydrogenation mainly consists of metal sites and support, including metal oxide/carbide, zeolite, graphene, and so on. Coperet and co-workers prepared zirconia-supported copper nanoparticles (NPs), which showed methanol selectivity of 75% (Larmier et al., 2017). Encapsulation of Cu NPs in Beta zeolite elevates ethanol selectivity and space-time yield (STY) to ∼ 100% and 398 mg g_cat_^−1^ h^−1^ (Ding et al., 2020). Highly selective conversion of CO_2_ into light olefins, aromatics, gasolines, and diesel was also achieved over metal oxides/zeolites bifunctional catalysts (Gao et al., 2017; Wang Y. et al., 2018; Zhou et al., 2019; Wang S. et al., 2020; Wei et al., 2021; Zhang et al., 2022b; Wang et al., 2022). In general, improvement of metal site dispersion and modulation of metal-support interaction can increase CO_2_ conversion and product selectivity.

In recent years, metal-organic frameworks (MOFs) have been considered important host materials in adsorption, gas separation and catalysis processes, due to their large surface area, abundant metal sites, and three-dimensional (3D) porous structure (Kumar et al., 2017; Guntern et al., 2021). MOFs are composed of metal-oxygen clusters serving as secondary building units (SBUs) that are connected by the organic ligands (Ranocchiari and Bokhoven, 2011; Chavan et al., 2012). The cages and the missing linker defects in MOFs are ideal places for confining ultra-small metal NPs, thus, inhibiting the sintering and aggregation of active sites (An et al., 2017; Zhao et al., 2018; Abdel-Mageed et al., 2019; Hu et al., 2019; Zhu et al., 2020; Yu et al., 2021). The metal-support interaction can be adjusted by controlling the pyrolysis of nodes and linkers in MOFs (Furukawa et al., 2010; Abdel-Mageed et al., 2019; Wang, 2022). This work gives a short review about the recent progress on the application of MOFs-based catalysts in CO_2_ hydrogenation to methanol, methane, and some C_2+_ products. Synthesis and structural regulation of MOFs materials have been reviewed in many other literatures (Eddaoudi et al., 2002; Cavka et al., 2008; Wang C. et al., 2018; Usman et al., 2021).

### CO_2_ Hydrogenation to Methanol

Methanol is an important platform compound in the chemical industry, and it can be transformed into commodity olefins, aromatics, formaldehyde, and longer-chain alcohols (Liu et al., 2018; Yarulina et al., 2018). Considering the unique features role of methanol in energy conversion, a concept of “methanol economy” was proposed by Olah and co-workers (Goeppert et al., 2014). Hydrogenation of CO_2_ to methanol is described as the following Equation 1

$$CO_{2}+3H_{2}=CH_{3}\mathrm{OH}+H_{2}O\;\Delta H_{298K}=-49.3kJ/mol$$ (1)

Although CO_2_ hydrogenation to methanol is exothermic, the activation of O=C=O bond requires to overcome a high energy barrier that makes operating temperature generally as high as 240–300°C (Murthy et al., 2021). An increase in reaction temperature not only accelerates competitive reverse water-gas shift (RWGS) reaction (Equation 2) and produces more CO but also induces sintering and aggregation of active metal NPs.

$$CO_{2}+H_{2}=\mathrm{CO}+H_{2}O\;\Delta H_{298K}=41.1kJ/mol$$ (2)

**FIGURE 1:**
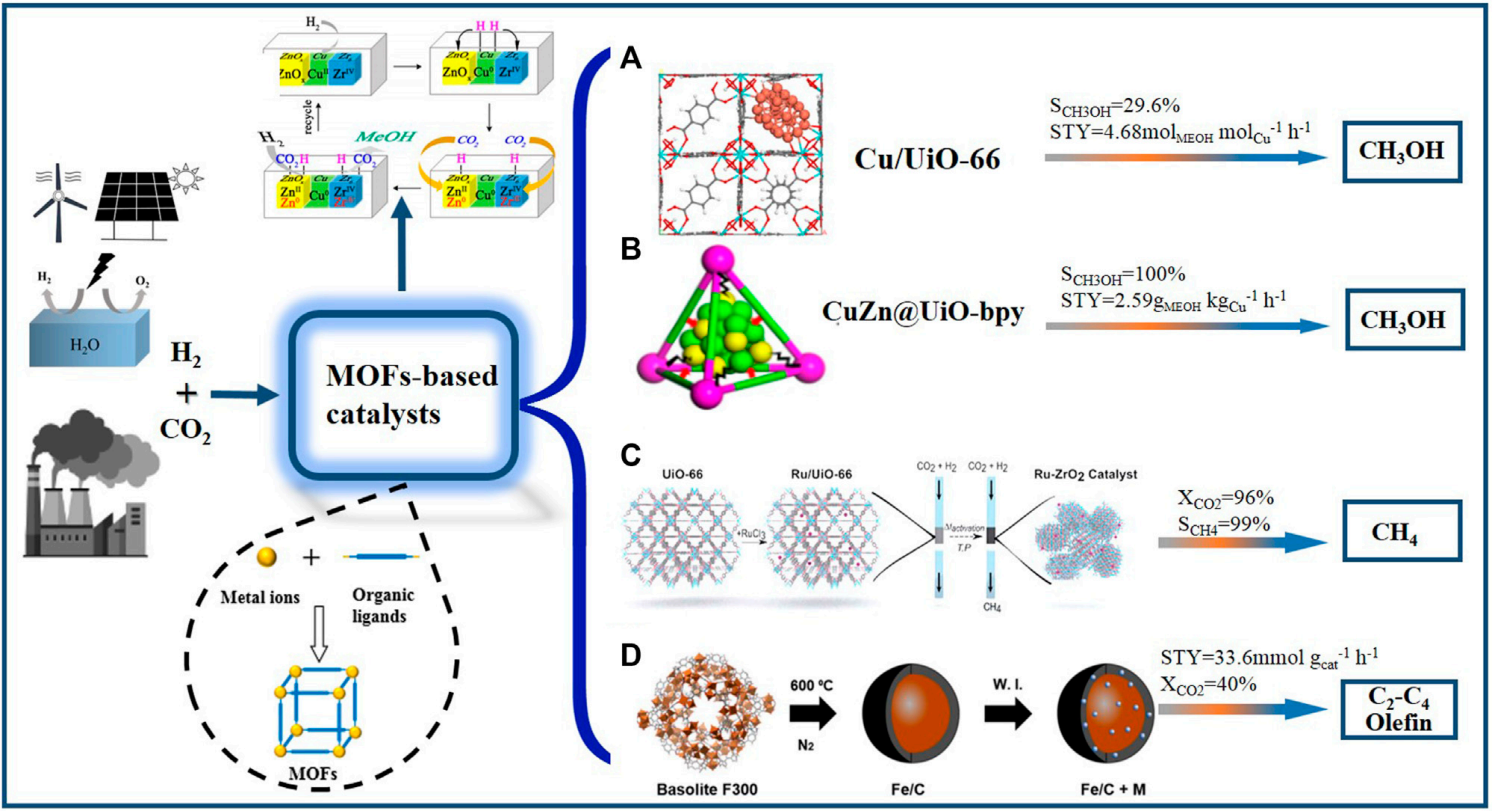
CO_2_ hydrogenation over various MOFs-based catalysts. **(A)** Interface bonding of sub-nanometer Cu clusters with Zr_6_O_8_ nodes over Cu/UiO-66 and its catalytic results in CO_2_ hydrogenation to methanol. Reproduced with permission from Zhu et al. (2020). Copyright 2020 Springer Nature. **(B)** The Cu/ZnO_x_ NPs embedded UiO-bpy and its catalytic results in CO_2_ hydrogenation to methanol. Reproduced with permission from An et al. (2017). Copyright 2017 American Chemical Society. **(C)** Structural evolution in the decomposition process of Ru/UiO-66 and its catalytic results obtained material for CO_2_ hydrogenation to CH_4_. Reproduced with permission from Lippi et al. (2017). Copyright 2017 Royal Society of Chemistry. **(D)** Fe/C-K catalyst fabricated through decomposition of Basolite F300 MOF and its catalytic results obtained material for CO_2_ hydrogenation to CH_4_. Reproduced with permission from Ramirez et al. (2018). Copyright 2018 American Chemical Society.

One efficient method to improve the sintering resistance of active metal species is the enhancement of metal-support interface interaction through confining ultrasmall metal NPs within the pores or cages of MOFs. Yaghi and co-workers prepared the catalyst with the single Cu nanocrystal encapsulated in the cage of UiO-66 (Cu@UiO-66); Cu@UiO-66 shows 100% methanol selectivity, and 8 times higher yield than Cu/ZnO/Al_2_O_3_ in CO_2_ hydrogenation (Rungtaweevoranit et al., 2016). It suggests that the strong interaction between Cu nanocrystal and Zr-based SBUs of UiO-66 effectively stabilizes Cu active sites. Anchoring Cu NPs into the missing-linker defects of UiO-66 considerably enhances the interaction of metallic Cu with Zr_6_O_8_ nodes of UiO-66. It is found that the isolated Cu can only produce CO via RWGS reaction, whereas Cu NPs anchored on the Zr_6_O_8_ nodes generates larger numbers of Cu-O-Zr interface sites that show higher activity for methanol synthesis (Figure 1A) (Zhu et al., 2020). An and co-workers reported that the organic coordinating groups in MOFs play a vital role in stabilizing metal NPs (An et al., 2017). The ultra-small Cu/ZnO_x_ NPs are *in situ* generated through the reduction of frameworks Cu^2+^ and Zn^2+^ ions in Zr_6_ clusters of UiO-bpy (2,2-bipyridine) MOF (Figure 1B). The strong interaction between Cu/ZnO_x_ NPs and bpy moieties in MOFs prevents the phase separation of Cu/ZnO_x_. Thus, the prepared Cu/ZnO_x_@MOFs catalyst shows methanol selectivity of 100% in CO_2_ hydrogenation, with the STY as high as 2.59 g_MeOH_ kg_Cu_^−1^ h^−1^. It should be noticed that although these MOF catalysts have been widely used in CO_2_ hydrogenation, they usually need to be carried out at a relatively low temperature, because of their low thermal/hydrothermal stability. It is found that the organic ligands in MOF decompose easily at the high reaction temperature, causing the collapse of the pore structure and the decrease of catalytic activity.

Another way to enhance the metal-support interaction is to pyrolyze the metal-loaded MOF precursors in an inert atmosphere. Liu and co-workers fabricated a stable Cu@ZrO_x_ catalyst via *in situ* treatment of Cu/UiO-66 in H_2_ flow at different temperatures (Liu et al., 2019). Cu@ZrO_x_ possesses abundant Cu-ZrO_x_ interfaces and a stable 3D ZrO_x_ framework that leads to the formation of more Cu^+^ species on the surface of ZrO_2_. As a result, CO_2_ hydrogenation to methanol is significantly improved via forming more formate and methoxy intermediates. An inverse ZnO/Cu catalyst with closer proximity to ZnO-Cu interface was prepared by Hu and co-workers through directly calcining Cu@ZIF-8 (Hu et al., 2019). It is shown that the small ZnO NPs on the surface of Cu promote the formation of methanol in CO_2_ hydrogenation. HKUST-1 was used as the Cu source to prepare the ZrO_2_@HKUST-1 core-shell precursor via one-step hydrothermal method. Upon calcination and reduction, the Cu nanoclusters are highly dispersed on ZrO_2_, forming strong Cu-ZrO_2_ interface interaction. This nano Cu-ZrO_2_ catalyst gives a methanol space-time yield (STY) of about 5.2 times higher than that of the sample obtained by the traditional impregnation method (Yu et al., 2021).

Besides Cu-based catalysts, other metals-loaded MOFs have been developed for CO_2_ hydrogenation to methanol. Yin and co-workers embedded ultrasmall Pd crystals into ZIF-8 and further pyrolyzed them into PdZn alloy after calcination under airflow (Yin et al., 2018). The strong interface interaction between PdZn and ZnO not only prevents the aggregation of metal sites but also leads to the formation of more oxygen defects, thereby enhancing catalytic activity and stability of PdZn catalyst in CO_2_ hydrogenation to methanol. Co_3_O_4_ coated by amorphous In_2_O_3_ shell was synthesized through decomposition of In-modified ZIF-67(Co) (Pustovarenko et al., 2020). Co_3_O_4_/In_2_O_3_ core-shell catalyst exhibits a maximum methanol STY of 0.65 g_MeOH_ g_cat_^−1^ h^−1^ over 100 h time on stream. Olsbye and co-workers (Gutterod et al., 2019) encapsulated Pt NPs into the octahedral cavity of UiO-67. The Pt-embedded UiO-67 produces more methanol but less methane in CO_2_ hydrogenation than the Pt/C, Pt/SiO_2_ and Pt/Al_2_O_3_ at 170°C and 1–8 bar. It is shown that the interface between Pt NPs and linker-deficient Zr_6_O_8_ nodes is the main site for methanol formation. A decrease in missing-linker defects lowered the methanol formation rate (Gutterød et al., 2020). Introduction of H_2_O is beneficial to increase in methanol selectivity, due to the facilitation of methanol desorption. The catalytic results of some MOFs-based catalysts in CO_2_ hydrogenation to methanol are summarized in Table 1.

**TABLE 1:** Summary performance of catalysts for CO_2_ hydrogenation reaction.

Catalysts	Main Product	H_2_/CO_2_ Ratio	T (^o^C)	P (MPa)	Loading (Wt%)	X_CO2_ (%)	Selectivity (%)	STY (gkg_cat_^−1^ h^−1^)	TOS (h)	Ref
Cu-UiO-66	Methanol	3:1	175	1	1.0	1	100	—	—	Rungtaweevoranit et al. (2016)
Cu/UiO-66	Methanol	3:1	250	3.2	1.4	-	29.6	679.76	50	Zhu et al. (2020)
Cu@3D-ZrOx	Methanol	3:1	260	4.5	12.4	13.1	78.8	796	105	Liu et al. (2019)
Cu/ZnO_x_@UiO-66	Methanol	3:1	250	4	5.9	4.3	87	28.3	100	Yang et al. (2021)
Cu/ZnO_x_@UiO-bpy	Methanol	3:1	250	4	6.9	3.3	100	37.5	100	An et al. (2017)
Cu-ZrO_2_(ZrO_2_@HKUST-1)	Methanol	3:1	220	3	11	6.8	64.4	287.9	16	Yu et al. (2021)
ZnO/Cu(Cu@ZIF-8)	Methanol	3:1	260	4.5	57.6	—	—	933	76	Hu et al. (2019)
PdZn (Cu@ZIF-8)	Methanol	3:1	270	4.5	—	14	55	650	50	Yin et al. (2018)
In_2_O_3_/Co_3_O_4_(In@ZIF-67)	Methanol	3:1	300	5	—	—	87	650	100	Pustovarenko et al. (2020)
Ni@MIL-101	Methane	8:1	320	0.1	10	56.4	91.6	—	10	Mihet et al. (2019)
Ni@MOF-5	Methane	4:1	320	0.1	10	75.1	100	—	100	Zhen et al. (2015)
Ni@UiO-66	Methane	3:1	300	—	20	57.6	100	—	100	Zhao et al. (2018)
Ru/UiO-66	Methane	4:1	350	0.5	1.0	96	99	—	—	Lippi et al. (2017)
K-CuZnAl + Na-Fe@C	Ethanol	3:1	350	5	—	39.2	35	—	50	Wang Y. et al., 2021
Fe/C-K (Basolite F300)	Olefins	3:1	320	3	—	40	∼40	—	50	Ramirez et al. (2018)

### CO_2_ Hydrogenation to Methane

Methane (CH_4_) is the main component of natural gas and it is also an important building block in the chemical industry, and can be further transformed into downstream products such as ethyne and ammonia (Bai et al., 2008; Sha et al., 2020). Hydrogenation of CO_2_ to CH_4_ provides an alternative solution to alleviated methane market shortage (Qin et al., 2017). Although CO_2_ methanation is a strongly exothermic reaction (Equation 3), it is always operated at high temperatures because of the kinetic limitation.

$$CO_{2}+4H_{2}=CH_{4}+2H_{2}O\Delta H_{298K}=-165.0kJ/mol$$ (3)

Supported ruthenium (Ru), rhodium (Rh), nickel (Ni), and cobalt (Co) catalysts are commonly used, as their high activity in CO_2_ hydrogenation. Strong metal-support interaction (SMSI) improves the dispersion of metal species and the stability of catalyst. Using SMSI to mediate the catalytic behavior of supported metal species is of significance in CO_2_ hydrogenation. MOFs are believed as promising supports or sacrificed templates, as they can promote active metal species dispersion and enhance the metal-support interaction.

Mihet and co-workers encapsulated Ni into MIL-101 (Ni@MIL-101) using the impregnation method. The high surface area (2,497 m^2^ g^−1^) and pore volume (1.75 cm^3^ g^−1^) of Ni@MIL-101 make the Ni particles highly dispersed in MIL-101 which enhances the adsorption and activation of CO_2_. With such a catalyst, CO_2_ conversion reaches 56.4%, with CH_4_ selectivity of 91.6% at 320°C and gas hourly space velocity (GHSV) of 4,650 ml g^−1^ h^−1^ (Mihet et al., 2019). Ni@MOF-5 shows higher Ni dispersion due to a larger surface area (2,961 m^2^ g^−1^) and thus resulting in higher CO_2_ conversion and CH_4_ selectivity of 75.1 and ∼100% respectively at 300°C, 1 atm and GHSV of 2000 ml g^−1^ h^−1^ (Zhen et al., 2015). In addition, the metal-support interaction is also enhanced by anchoring Ni on MOFs. Zhao and co-workers prepared a series of UiO-66-supported Ni-based catalysts using impregnation and reduction methods (Zhao et al., 2018). Encapsulation of ultrasmall Ni particles in UiO-66 can increase the interface interaction of Ni with UiO-66 support, which, hence, inhibits the sintering of Ni species. The prepared 20%Ni@UiO-66 exhibits CO_2_ conversion of 57.6% and CH_4_ selectivity of 100% in CO_2_ hydrogenation. Interestingly, no significant deactivation was observed even after a reaction of 100 h.

Lippi and co-workers investigated the structural evolution in the decomposition process of metal-loaded MOFs (Lippi et al., 2017; Lippi et al., 2021). The 3D framework of Ru-impregnated UiO-66 (Ru/UiO-66) gradually collapsed under CO_2_ methanation conditions to form amorphous C and Zr containing phase structure, which is then transformed into tetragonal ZrO_2_(t) and finally into more stable monoclinic ZrO_2_(m) (Figure 1C). The structure and morphology of catalyst can be precisely controlled by altering the treatment conditions during MOFs decomposition. The Ru/ZrO_2_(m) is a highly active and stable CO_2_ methanation catalyst and it gives the CO_2_ conversion of 96% and CH_4_ selectivity of 99% at 350°C and 5 bar. The catalytic results of reported MOFs-based catalysts for CO_2_ hydrogenation to CH_4_ was shown in Table 1.

### CO_2_ Hydrogenation to C_2+_ Products

Conversion of CO_2_ to C_2+_ products, such as alcohol, olefins and aromatics, is highly desirable but remains a greater challenge than to C1 compounds due to the high C-C coupling barrier (Guo et al., 2018; Wei et al., 2021). Although CO_2_ hydrogenation to ethanol, light olefin, or aromatic is exothermic (Equations 4–6), relatively high temperature and pressure are always necessary for the activation of CO_2_ molecules.

$$2CO_{2}+6H_{2}=C_{2}H_{5}\mathrm{OH}+3H_{2}O\Delta H_{298K}=-173.3kJ/mol$$ (4)

$$2CO_{2}+6H_{2}=C_{2}H_{4}+4H_{2}O\Delta H_{298K}=-127.9kJ/mol$$ (5)

$$6CO_{2}+15H_{2}=C_{6}H_{6}+12H_{2}O\Delta H_{298K}=-457.9kJ/mol$$ (6)

Recently, some researchers suggested that direct pyrolysis of metal-loaded MOFs in an inert atmosphere can generate various metal-carbides, in which the metal NPs are closely confined in the carbon porous materials to achieve strong metal-support interaction and avoid aggregation of active sites. Tsubaki and co-workers designed the K-CuZnAl and Na-Fe@C composite catalyst. This catalyst enhances the ethanol selectivity as high as 35.0% and CO_2_ conversion of 39.2%, at 350°C and 5.0 MPa in CO_2_ hydrogenation (Wang Y. et al., 2021). The K-CuZnAl activates CO_2_ to form methanol and CO, and Na-Fe@C promotes the C-C coupling, with Na-Fe@C obtained from the pyrolysis of Fe-based MOFs under N_2_ flow. The carbon matrix effectively prevents Fe sintering and achieves the uniform dispersion of Fe active sites.

The hydrogenation of CO_2_ to olefins is a potential way of achieving a sustainable carbon cycle. It is generally performed via a two-step process on Fe-based catalysts: CO_2_ is firstly converted to CO via RWGS reaction, and then CO is hydrogenated to olefins via Fischer-Tropsch synthesis (FTS) reaction (Yang et al., 2017; De et al., 2020). Ramirez and co-workers prepared the Fe/C-K catalyst through the decomposition of Basolite F300 MOF under N_2_ atmosphere (Ramirez et al., 2018). It shows good catalytic performance for CO_2_ hydrogenation to olefins; CO_2_ conversion and C_2_^=^-C_4_^=^ STY reach 40% and 33.6 mmol g_cat_^−1^ h^−1^ at 320°C and 3 MPa (Figure 1D). The uniform distribution of Fe active sites is considered effectively promote RWGS and FTS reactions.

Similarly, Li and co-workers fabricated ZnZrO_x_@C catalyst with three-dimensional (3D) hierarchical structure through carbonization of Zn-modified UiO-66 (Wang Y. et al., 2020). The introduction of Zn into the synthesis gel of UiO-66 induces the formation of more defects due to the substitution of Zn for Zr. Upon coupling with H-ZSM-5 zeolite, it affords the selectivity of aromatics in hydrocarbons as high as 73.1%, CH_4_ is decreased to 3.4%. Compared with possesses traditional ZnZrO_x_ oxides, ZnZrO_x_@C catalyst formed by the carbonization of defective MOFs owns richer surface vacancies, and hence, shows higher CO/CO_2_ conversion due to their strong adsorption. In addition, the 3D hierarchical carbon framework structure facilitates the diffusion of products, thus, avoiding secondary reactions and elevating the proportion of benzene, toluene, and xylene (BTX) in aromatics.

## Discussion

Metal-organic frameworks (MOFs) are burgeoning porous materials and they are widely used in adsorption, separation, and catalysis processes due to their unique pore structure, versatile compositions, and large surface area. The cages and the missing-linker defects in MOFs provide ideal places for encapsulating or anchoring metal nanoparticles, thereby preventing the sintering and aggregation of active sites. Nevertheless, MOFs are also important sacrificed templates for the preparation of high-efficient metal oxides or carbides. Some MOFs derived metal@C were designed, and exhibited high catalytic performance in CO_2_ hydrogenation to methanol, methane, and other C_2+_ products, due to high dispersion of active sites and strong metal-support interaction.

Despite that MOFs as supports or catalysts have received extensive attention and they show superior catalytic activity and product selectivity in CO_2_ hydrogenation, however, the much lower thermal and hydrothermal stability of MOFs than SiO_2_, Al_2_O_3,_ and zeolites limits the applications in many industrial processes. This is because the organic ligands are nearly impossible to resist high temperature or their facile pyrolysis character. Nevertheless, there are potential candidates for preparing highly dispersed oxide-supported metal catalysts with strong metal-support interaction.

## References

[B1] Abdel-Mageed A. M. Rungtaweevoranit B. Parlinska-Wojtan M. Pei X. Yaghi O. M. Behm R. J. (2019). Highly Active and Stable Single-Atom Cu Catalysts Supported by a Metal-Organic Framework. J. Am. Chem. Soc. 141, 5201–5210. 10.1021/jacs.8b11386 30852893

[B2] An B. Zhang J. Cheng K. Ji P. Wang C. Lin W. (2017). Confinement of Ultrasmall Cu/ZnOx Nanoparticles in Metal-Organic Frameworks for Selective Methanol Synthesis from Catalytic Hydrogenation of CO_2_ . J. Am. Chem. Soc. 139, 3834–3840. 10.1021/jacs.7b00058 28209054

[B3] Bai M. Zhang Z. Bai M. Bai X. Gao H. (2008). Synthesis of Ammonia Using CH4/N2 Plasmas Based on Micro-Gap Discharge under Environmentally Friendly Condition. Plasma Chem. Plasma Process 28, 405–414. 10.1007/s11090-008-9132-4

[B4] Cavka J. H. Jakobsen S. Olsbye U. Guillou N. Lamberti C. Bordiga S. (2008). A New Zirconium Inorganic Building Brick Forming Metal Organic Frameworks with Exceptional Stability. J. Am. Chem. Soc. 130, 13850–13851. 10.1021/ja8057953 18817383

[B5] Chavan S. Vitillo J. G. Gianolio D. Zavorotynska O. Civalleri B. Jakobsen S. (2012). H2 Storage in Isostructural UiO-67 and UiO-66 MOFs. Phys. Chem. Chem. Phys. 14, 1614–1626. 10.1039/c1cp23434j 22187720

[B6] De S. Dokania A. Ramirez A. Gascon J. (2020). Advances in the Design of Heterogeneous Catalysts and Thermocatalytic Processes for CO_2_ Utilization. ACS Catal. 10, 14147–14185. 10.1021/acscatal.0c04273

[B7] Didas S. A. Choi S. Chaikittisilp W. Jones C. W. (2015). Amine-Oxide Hybrid Materials for CO_2_ Capture from Ambient Air. Acc. Chem. Res. 48, 2680–2687. 10.1021/acs.accounts.5b00284 26356307

[B8] Ding L. Shi T. Gu J. Cui Y. Zhang Z. Yang C. (2020). CO_2_ Hydrogenation to Ethanol over Cu@Na-Beta. Chem 6, 2673–2689. 10.1016/j.chempr.2020.07.001

[B9] Eddaoudi M. Kim J. Rosi N. Vodak D. Wachter J. O'Keeffe M. (2002). Systematic Design of Pore Size and Functionality in Isoreticular MOFs and Their Application in Methane Storage. Science 295, 469–472. 10.1126/science.1067208 11799235

[B10] Furukawa H. Ko N. Go Y. B. Aratani N. Choi S. B. Choi E. (2010). Ultrahigh Porosity in Metal-Organic Frameworks. Science 329, 424–428. 10.1126/science.1192160 20595583

[B11] Gao P. Dang S. Li S. Bu X. Liu Z. Qiu M. (2017). Direct Production of Lower Olefins from CO_2_ Conversion via Bifunctional Catalysis. ACS Catal. 8, 571–578. 10.1021/acscatal.7b02649

[B12] Goeppert A. Czaun M. Jones J.-P. Surya Prakash G. K. Olah G. A. (2014). Recycling of Carbon Dioxide to Methanol and Derived Products - Closing the Loop. Chem. Soc. Rev. 43, 7995–8048. 10.1039/c4cs00122b 24935751

[B13] Guntern Y. T. Vávra J. Karve V. V. Varandili S. B. Segura Lecina O. Gadiyar C. (2021). Synthetic Tunability of Colloidal Covalent Organic Framework/nanocrystal Hybrids. Chem. Mat. 33, 2646–2654. 10.1021/acs.chemmater.1c00501

[B14] Guo L. Sun J. Ge Q. Tsubaki N. (2018). Recent Advances in Direct Catalytic Hydrogenation of Carbon Dioxide to Valuable C_2+_ Hydrocarbons. J. Mat. Chem. A 6, 23244–23262. 10.1039/c8ta05377d

[B15] Gutterød E. S. Pulumati S. H. Kaur G. Lazzarini A. Solemsli B. G. Gunnæs A. E. (2020). Influence of Defects and H2O on the Hydrogenation of CO_2_ to Methanol over Pt Nanoparticles in UiO-67 Metal-Organic Framework. J. Am. Chem. Soc. 142, 17105–17118. 10.1021/jacs.0c07153 32902970 PMC7586342

[B16] Gutterod E. S. Lazzarini A. Fjermestad T. Kaur G. Manzoli M. Bordiga S. (2019). Hydrogenation of CO_2_ to Methanol by Pt Nanoparticles Encapsulated in UiO-67: Deciphering the Role of the Metal–Organic Framework. J. Am. Chem. Soc. 142, 999–1009. 10.1021/jacs.9b10873 31794194

[B17] Handoko A. D. Li K. Tang J. (2013). Recent Progress in Artificial Photosynthesis: CO_2_ Photoreduction to Valuable Chemicals in a Heterogeneous System. Curr. Opin. Chem. Eng. 2, 200–206. 10.1016/j.coche.2012.12.003

[B18] Hu B. Yin Y. Zhong Z. Wu D. Liu G. Hong X. (2019). Cu@ZIF-8 Derived Inverse ZnO/Cu Catalyst with Sub-5 Nm ZnO for Efficient CO_2_ Hydrogenation to Methanol. Catal. Sci. Technol. 9, 2673–2681. 10.1039/c8cy02546k

[B19] Jhong H.-R. M. Ma S. Kenis P. J. (2013). Electrochemical Conversion of CO_2_ to Useful Chemicals: Current Status, Remaining Challenges, and Future Opportunities. Curr. Opin. Chem. Eng. 2, 191–199. 10.1016/j.coche.2013.03.005

[B20] Kumar P. Vellingiri K. Kim K.-H. Brown R. J. C. Manos M. J. (2017). Modern Progress in Metal-Organic Frameworks and Their Composites for Diverse Applications. Microporous Mesoporous Mater. 253, 251–265. 10.1016/j.micromeso.2017.07.003

[B21] Larmier K. Liao W.-C. Tada S. Lam E. Verel R. Bansode A. (2017). CO_2_-to-Methanol Hydrogenation on Zirconia-Supported Copper Nanoparticles: Reaction Intermediates and the Role of the Metal-Support Interface. Angew. Chem. Int. Ed. 56, 2318–2323. 10.1002/anie.201610166 28111850

[B22] Lippi R. D’Angelo A. M. Li C. Howard S. C. Madsen I. C. Wilson K. (2021). Unveiling the Structural Transitions during Activation of a CO_2_ Methanation Catalyst Ru0/ZrO2 Synthesised from a MOF Precursor. Catal. Today 368, 66–77. 10.1016/j.cattod.2020.04.043

[B23] Lippi R. Howard S. C. Barron H. Easton C. D. Madsen I. C. Waddington L. J. (2017). Highly Active Catalyst for CO_2_ Methanation Derived from a Metal Organic Framework Template. J. Mat. Chem. A 5, 12990–12997. 10.1039/c7ta00958e

[B24] Liu L. Zhao H. Andino J. M. Li Y. (2012). Photocatalytic CO_2_ Reduction with H_2_O on TiO_2_ Nanocrystals: Comparison of Anatase, Rutile, and Brookite Polymorphs and Exploration of Surface Chemistry. ACS Catal. 2, 1817–1828. 10.1021/cs300273q

[B25] Liu T. Hong X. Liu G. (2019). *In Situ* Generation of the Cu@3D-ZrOx Framework Catalyst for Selective Methanol Synthesis from CO_2_/H_2_ . ACS Catal. 10, 93–102. 10.1021/acscatal.9b03738

[B26] Liu W.-C. Baek J. Somorjai G. A. (2018). The Methanol Economy: Methane and Carbon Dioxide Conversion. Top. Catal. 61, 530–541. 10.1007/s11244-018-0907-4

[B27] Mihet M. Grad O. Blanita G. Radu T. Lazar M. D. (2019). Effective Encapsulation of Ni Nanoparticles in Metal-Organic Frameworks and Their Application for CO_2_ Methanation. Int. J. Hydrogen Energy 44, 13383–13396. 10.1016/j.ijhydene.2019.03.259

[B28] Murthy P. S. Liang W. Jiang Y. Huang J. (2021). Cu-Based Nanocatalysts for CO_2_ Hydrogenation to Methanol. Energy fuels. 35, 8558–8584. 10.1021/acs.energyfuels.1c00625

[B29] NOAA (2022). Trends in Atmospheric Carbon Dioxide: Full Record. ESRL Global Monitoring Division, Global Greenhouse Gas Reference Network. Available at: https://gml.noaa.gov/ccgg/trends/global.html (Accessed Apr 15, 2022).

[B30] Perathoner S. Centi G. (2019). Catalysis for Solar-Driven Chemistry: The Role of Electrocatalysis. Catal. Today 330, 157–170. 10.1016/j.cattod.2018.03.005

[B31] Porosoff M. D. Yan B. Chen J. G. (2016). Catalytic Reduction of CO_2_ by H_2_ for Synthesis of CO, Methanol and Hydrocarbons: Challenges and Opportunities. Energy Environ. Sci. 9, 62–73. 10.1039/c5ee02657a

[B32] Pustovarenko A. Dikhtiarenko A. Bavykina A. Gevers L. Ramírez A. Russkikh A. (2020). Metal-Organic Framework-Derived Synthesis of Cobalt Indium Catalysts for the Hydrogenation of CO_2_ to Methanol. ACS Catal. 10, 5064–5076. 10.1021/acscatal.0c00449

[B33] Qin Z. Zhou Y. Jiang Y. Liu Z. Ji H. (2017). “Recent Advances in Heterogeneous Catalytic Hydrogenation of CO_2_ to Methane,” in New Advances in Hydrogenation Processes - Fundamentals and Applications (Londan: IntechOpen), 57–82.

[B34] Rafiee A. Rajab Khalilpour K. Milani D. Panahi M. (2018). Trends in CO_2_ Conversion and Utilization: A Review from Process Systems Perspective. J. Environ. Chem. Eng. 6, 5771–5794. 10.1016/j.jece.2018.08.065

[B35] Ramirez A. Gevers L. Bavykina A. Ould-Chikh S. Gascon J. (2018). Metal Organic Framework-Derived Iron Catalysts for the Direct Hydrogenation of CO_2_ to Short Chain Olefins. ACS Catal. 8, 9174–9182. 10.1021/acscatal.8b02892

[B36] Ranocchiari M. Bokhoven J. A. v. (2011). Catalysis by Metal–Organic Frameworks: Fundamentals and Opportunities. Phys. Chem. Chem. Phys. 13, 6388. 10.1039/c0cp02394a 21234497

[B37] Rungtaweevoranit B. Baek J. Araujo J. R. Archanjo B. S. Choi K. M. Yaghi O. M. (2016). Copper Nanocrystals Encapsulated in Zr-Based Metal-Organic Frameworks for Highly Selective CO_2_ Hydrogenation to Methanol. Nano Lett. 16, 7645–7649. 10.1021/acs.nanolett.6b03637 27960445

[B38] Sha F. Han Z. Tang S. Wang J. Li C. (2020). Hydrogenation of Carbon Dioxide to Methanol over Non-cu-based Heterogeneous Catalysts. ChemSusChem 13, 6160–6181. 10.1002/cssc.202002054 33146940

[B39] Song C. (2006). Global Challenges and Strategies for Control, Conversion and Utilization of CO_2_ for Sustainable Development Involving Energy, Catalysis, Adsorption and Chemical Processing. Catal. Today 115, 2–32. 10.1016/j.cattod.2006.02.029

[B40] Srinivas G. Krungleviciute V. Guo Z.-X. Yildirim T. (2014). Exceptional CO_2_capture in a Hierarchically Porous Carbon with Simultaneous High Surface Area and Pore Volume. Energy Environ. Sci. 7, 335–342. 10.1039/c3ee42918k

[B41] Usman M. Helal A. Abdelnaby M. M. Alloush A. M. Zeama M. Yamani Z. H. (2021). Trends and Prospects in UiO‐66 Metal‐Organic Framework for CO 2 Capture, Separation, and Conversion. Chem. Rec. 21, 1771–1791. 10.1002/tcr.202100030 33955166

[B42] Valles-Regino R. Tate R. Kelaher B. Savins D. Dowell A. Benkendorff K. (2015). Ocean Warming and CO_2_-Induced Acidification Impact the Lipid Content of a Marine Predatory Gastropod. Mar. Drugs 13, 6019–6037. 10.3390/md13106019 26404318 PMC4626677

[B43] Wang C. An B. Lin W. (2018). Metal-Organic Frameworks in Solid-Gas Phase Catalysis. ACS Catal. 9, 130–146. 10.1021/acscatal.8b04055

[B44] Wang H. (2022). Nanostructure@metal-organic Frameworks (MOFs) for Catalytic Carbon Dioxide (CO_2_) Conversion in Photocatalysis, Electrocatalysis, and Thermal Catalysis. Nano Res. 15, 2834–2854. 10.1007/s12274-021-3984-9

[B45] Wang J.-J. Li X.-P. Cui B.-F. Zhang Z. Hu X.-F. Ding J. (2021). A Review of Non-noble Metal-Based Electrocatalysts for CO_2_ Electroreduction. Rare Met. 40, 3019–3037. 10.1007/s12598-021-01736-x

[B46] Wang S. Zhang L. Wang P. Liu X. Chen Y. Qin Z. (2022). Highly Effective Conversion of CO_2_ into Light Olefins Abundant in Ethene. Chem 8, 1376–1394. 10.1016/j.chempr.2022.01.004

[B47] Wang S. Zhang L. Zhang W. Wang P. Qin Z. Yan W. (2020). Selective Conversion of CO_2_ into Propene and Butene. Chem 6, 3344–3363. 10.1016/j.chempr.2020.09.025

[B48] Wang W.-H. Himeda Y. Muckerman J. T. Manbeck G. F. Fujita E. (2015). CO_2_ Hydrogenation to Formate and Methanol as an Alternative to Photo- and Electrochemical CO_2_ Reduction. Chem. Rev. 115, 12936–12973. 10.1021/acs.chemrev.5b00197 26335851

[B49] Wang Y. Tan L. Tan M. Zhang P. Fang Y. Yoneyama Y. (2018). Rationally Designing Bifunctional Catalysts as an Efficient Strategy to Boost CO_2_ Hydrogenation Producing Value-Added Aromatics. ACS Catal. 9, 895–901. 10.1021/acscatal.8b01344

[B50] Wang Y. Wang K. Zhang B. Peng X. Gao X. Yang G. (2021). Direct Conversion of CO_2_ to Ethanol Boosted by Intimacy-Sensitive Multifunctional Catalysts. ACS Catal. 11, 11742–11753. 10.1021/acscatal.1c01504

[B51] Wang Y. Zhan W. Chen Z. Chen J. Li X. Li Y. (2020). Advanced 3D Hollow-Out ZnZrO@C Combined with Hierarchical Zeolite for Highly Active and Selective CO Hydrogenation to Aromatics. ACS Catal. 10, 7177–7187. 10.1021/acscatal.0c01418

[B52] Wei J. Yao R. Han Y. Ge Q. Sun J. (2021). Towards the Development of the Emerging Process of CO_2_ Heterogenous Hydrogenation into High-Value Unsaturated Heavy Hydrocarbons. Chem. Soc. Rev. 50, 10764–10805. 10.1039/d1cs00260k 34605829

[B53] Yang H. Zhang C. Gao P. Wang H. Li X. Zhong L. (2017). A Review of the Catalytic Hydrogenation of Carbon Dioxide into Value-Added Hydrocarbons. Catal. Sci. Technol. 7, 4580–4598. 10.1039/c7cy01403a

[B54] Yang Y. Xu Y. Ding H. Yang D. Cheng E. Hao Y. (2021). Cu/ZnOx@UiO-66 Synthesized from a Double Solvent Method as an Efficient Catalyst for CO_2_ Hydrogenation to Methanol. Catal. Sci. Technol. 11, 4367–4375. 10.1039/d0cy02450c

[B55] Yarulina I. Chowdhury A. D. Meirer F. Weckhuysen B. M. Gascon J. (2018). Recent Trends and Fundamental Insights in the Methanol-To-Hydrocarbons Process. Nat. Catal. 1, 398–411. 10.1038/s41929-018-0078-5

[B56] Yin Y. Hu B. Li X. Zhou X. Hong X. Liu G. (2018). Pd@zeolitic Imidazolate Framework-8 Derived PdZn Alloy Catalysts for Efficient Hydrogenation of CO_2_ to Methanol. Appl. Catal. B Environ. 234, 143–152. 10.1016/j.apcatb.2018.04.024

[B57] Yu J. Liu S. Mu X. Yang G. Luo X. Lester E. (2021). Cu-ZrO2 Catalysts with Highly Dispersed Cu Nanoclusters Derived from ZrO2@ HKUST-1 Composites for the Enhanced CO_2_ Hydrogenation to Methanol. Chem. Eng. J. 419, 129656. 10.1016/j.cej.2021.129656

[B58] Zhang W. Jin Z. Chen Z. (2022a). Rational‐Designed Principles for Electrochemical and Photoelectrochemical Upgrading of CO_2_ to Value‐Added Chemicals. Adv. Sci. 9, 2105204. 10.1002/advs.202105204 PMC8948570 35072349

[B59] Zhang W. Ma D. Pérez-Ramírez J. Chen Z. (2021). Recent Progress in Materials Exploration for Thermocatalytic, Photocatalytic, and Integrated Photothermocatalytic CO 2 ‐to‐Fuel Conversion. Adv Energy Sustain Res 3, 2100169. 10.1002/aesr.202100169

[B60] Zhang W. Wang S. Guo S. Qin Z. Dong M. Wang J. (2022b). Effective Conversion of CO_2_ into Light Olefins over a Bifunctional Catalyst Consisting of La-Modified ZnZrOx Oxide and Acidic Zeolite. Catal. Sci. Technol. 12, 2566–2577. 10.1039/d2cy00210h

[B61] Zhao Z.-W. Zhou X. Liu Y.-N. Shen C.-C. Yuan C.-Z. Jiang Y.-F. (2018). Ultrasmall Ni Nanoparticles Embedded in Zr-Based MOFs Provide High Selectivity for CO_2_ Hydrogenation to Methane at Low Temperatures. Catal. Sci. Technol. 8, 3160–3165. 10.1039/c8cy00468d

[B62] Zhen W. Li B. Lu G. Ma J. (2015). Enhancing Catalytic Activity and Stability for CO_2_ Methanation on Ni@MOF-5 via Control of Active Species Dispersion. Chem. Commun. 51, 1728–1731. 10.1039/c4cc08733j 25518948

[B63] Zhou C. Shi J. Zhou W. Cheng K. Zhang Q. Kang J. (2019). Highly Active ZnO-ZrO2 Aerogels Integrated with H-ZSM-5 for Aromatics Synthesis from Carbon Dioxide. ACS Catal. 10, 302–310. 10.1021/acscatal.9b04309

[B64] Zhu Y. Zheng J. Ye J. Cui Y. Koh K. Kovarik L. (2020). Copper-zirconia Interfaces in UiO-66 Enable Selective Catalytic Hydrogenation of CO_2_ to Methanol. Nat. Commun. 11, 5849. 10.1038/s41467-020-19438-w 33208734 PMC7674450

